# Ecological Implications of a Flower Size/Number Trade-Off in Tropical Forest Trees

**DOI:** 10.1371/journal.pone.0016111

**Published:** 2011-02-01

**Authors:** Chris J. Kettle, Colin R. Maycock, Jaboury Ghazoul, Pete M. Hollingsworth, Eyen Khoo, Rahayu Sukmaria Haji Sukri, David F. R. P. Burslem

**Affiliations:** 1 Ecosystem Management, Institute of Terrestrial Ecosystems, ETH Zurich, Zurich, Switzerland; 2 Institute of Biological and Environmental Sciences, University of Aberdeen, Aberdeen, United Kingdom; 3 Royal Botanic Gardens Edinburgh, Edinburgh, United Kingdom; 4 Forest Research Centre, Sabah Forest Department, Sabah, Malaysia; Centre National de la Recherche Scientifique, France

## Abstract

**Background:**

In angiosperms, flower size commonly scales negatively with number. The ecological consequences of this trade-off for tropical trees remain poorly resolved, despite their potential importance for tropical forest conservation. We investigated the flower size number trade-off and its implications for fecundity in a sample of tree species from the Dipterocarpaceae on Borneo.

**Methodology/Principal Findings:**

We combined experimental exclusion of pollinators in 11 species, with direct and indirect estimates of contemporary pollen dispersal in two study species and published estimates of pollen dispersal in a further three species to explore the relationship between flower size, pollinator size and mean pollen dispersal distance. Maximum flower production was two orders of magnitude greater in small-flowered than large-flowered species of Dipterocarpaceae. In contrast, fruit production was unrelated to flower size and did not differ significantly among species. Small-flowered species had both smaller-sized pollinators and lower mean pollination success than large-flowered species. Average pollen dispersal distances were lower and frequency of mating between related individuals was higher in a smaller-flowered species than a larger-flowered confamilial. Our synthesis of pollen dispersal estimates across five species of dipterocarp suggests that pollen dispersal scales positively with flower size.

**Conclusions and Their Significance:**

Trade-offs embedded in the relationship between flower size and pollination success contribute to a reduction in the variance of fecundity among species. It is therefore plausible that these processes could delay competitive exclusion and contribute to maintenance of species coexistence in this ecologically and economically important family of tropical trees. These results have practical implications for tree species conservation and restoration. Seed collection from small-flowered species may be especially vulnerable to cryptic genetic erosion. Our findings also highlight the potential for differential vulnerability of tropical tree species to the deleterious consequences of forest fragmentation.

## Introduction

Angiosperms manifest a wide array of floral displays, from species that produce only a single large flower per plant, to those that produce hundreds of tiny flowers on many inflorescences per individual. The attractiveness of these different floral displays is closely coupled to mating system, the type and breadth of pollinators and individual fecundity [1], [2]. Studies across plant species show that species tend to produce fewer flowers per individual as the size of the flower increases [3], [4], [5], [6], [7],[8],[9]. The causal agents of this trade-off have been investigated empirically in a number of plant species, indicating that hierarchical resource allocation [10], [11], pollen discounting via geitonogamy in obligate out-crossing species [1], [4], [11] and pollen limitation [12] are all important drivers in this trade-off.

Few studies have, however, determined whether a flower size/number trade-off occurs across, as well as within, species (but see 7) and examined its consequences for plant community structure. The potential implications could include partitioning of pollinators among species as a function of flower size, differential pollen dispersal as a function of pollinator size and mobility relationships [13], and differences in fecundity among species. Surprisingly, interactions between plants and their pollinators have received limited attention as a driver of species diversity in tropical tree communities [14], [15], [16]. Partitioning of pollinator services could limit competitive exclusion, and if these interactions can be shown to contribute to an equalisation of fitness, contribute to species coexistence [17].

We combine experimental ecology, field observation and molecular ecological approaches to investigate the ecological implications of a flower size/number trade-off in a clade of tropical forest canopy and emergent trees. The processes that determine the differential fecundity of individuals are poorly understood, and therefore the importance to demography of pre-dispersal stages of plant reproduction may have been underestimated. Studies that link floral traits to fecundity are also likely to reveal important implications of flower size vs number trade-offs for species' differential responses to habitat degradation [12], [18]. For example, increased isolation of individual trees is of concern for the management of tropical tree species that have been heavily fragmented in recent years [19].

We used a group of 12 coexisting dipterocarp species ranging in flower size (calyx diameter from 1.2 mm to 10.2 mm) to explore the function of flower size and number and pollination systems in fruit production. Specifically, we combine measures of flower size, flower number and pollination success (as inferred from pollen tube growth data) with measures of paternity and genetic relatedness within mapped populations of two species to test the following explicit hypotheses.

1. Across species, flower size scales negatively with mean flower production per individual, which would be indicative of a flower size/number trade-off.
2. Across species, mean pollination success (as defined by the proportion of flower styles containing pollen tubes) increases with flower size.
3. Species with small flowers have small-bodied pollinators.
4. Species with smaller bodied pollinators have more limited pollen dispersal distances.
5. The proportion of flowers that give rise to fruit scales positively with flower size.
6. Spatial aggregation and limited pollen dispersal increase inbreeding in a small flowered species.

**Figure 1:**
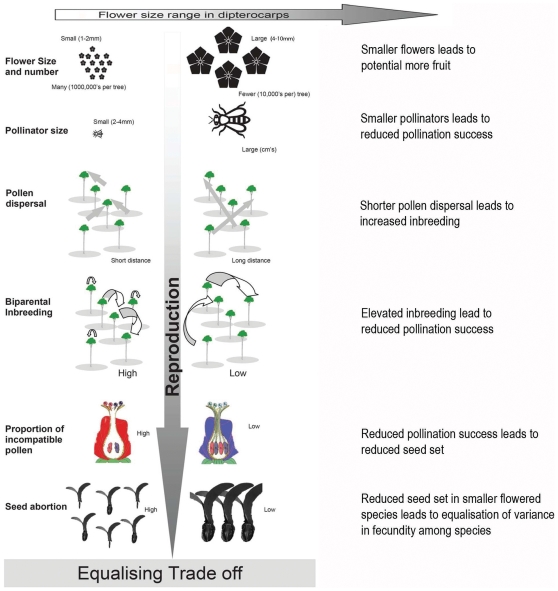
Schematic illustrating how interactions between flower size and number, pollination biology, pollen dispersal and fruit production might contribute to equalisation of fitness components among species of Dipterocarpaceae.

These hypotheses encapsulate a set of trade-offs between flower size, pollinator body size and pollen dispersal that might have consequences for fruit production and fecundity, which are important components of plant fitness (see Figure 1).

Tree species in the Dipterocarpaceae dominate lowland rain forests of Southeast Asia, accounting for up to 80 percent of the density and basal area of canopy trees [20]. Dipterocarps display variable flower size and morphology and are pollinated by insects including thrips, beetles, moths and giant honeybees [21], [22], [23], [24], [25], [26], [27]. Reproductively mature trees of many dipterocarps exhibit irregular mass flowering [20]. Dipterocarps produce hermaphroditic flowers and are considered predominately out-crossing, which is maintained through pre- and post- zygotic incompatibility mechanisms [28]. Apomixis has been reported in some species [24], [29], but we found no evidence of this in the progeny arrays of our study species. Fruit dispersal occurs by gravity and wind assisted gyration for the majority of fruit falling beneath the mother tree or a within 20–30 m [30], [31]. Limited seed dispersal leads to the aggregation of related individuals, and significant fine-scale spatial genetic structure has been observed in the majority of dipterocarp species studied [32].

## Results

### Flower and fruit production as a function of flower size

**Figure 2:**
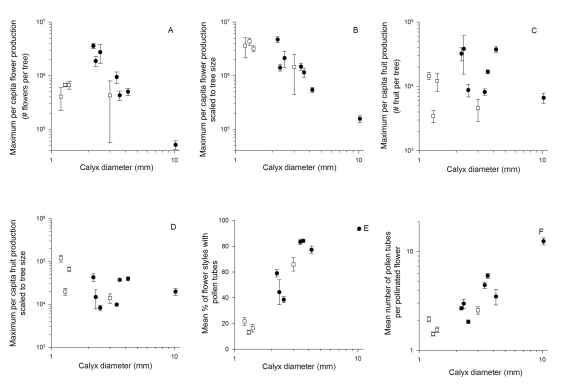
Effects of calyx tube diameter (flower size, mm) on estimated flower and fruit production in 11 dipterocarp tree species. Effects of calyx tube diameter on estimated (a) maximum per capita flower production, (b) maximum per capita flower production scaled to basal area and mean species tree height, (c) maximum per capita fruit production, (d) maximum per capita fruit production scaled to basal area and mean species tree height, (e) mean percentage of flower styles with pollen tubes and (f) mean number of pollen tubes per pollinated flower for four canopy (open symbols) and seven emergent (closed symbols) dipterocarp species growing in Sepilok Forest Reserve, Sabah. Maximum flower and fruit production were estimated from the mean of the upper quartile of values per species (n = 2 to 14 individuals per species).

Analyses of absolute (unscaled) log-transformed values of maximum potential flower production yielded a single most likely model (Table S1a Supplementary Information), in which flower production declined as a function of flower size for the comparison of 11 species (F_1,7_ = 35.88, *P* = 0.0005, Fig. 2a). In this model canopy species had lower values of unscaled maximum flower production than emergent species (F_1,7_ = 57.05, *P* = 0.0001), and the interaction between life form and flower size was also significant (F_1,7_ = 21.81, *P* = 0.0023). Analyses of maximum flower production scaled to tree size yielded three most likely models (Table S1a Supplementary Information). In all three cases scaled maximum flower production declined significantly with increasing flower size (e.g. F_1,7_ = 70.39, *P*<0.0001 for the model with the lowest AIC value, Fig. 2b)), but there was no difference between canopy and emergent species (F_1,7_ = 1.71, *P* = 0.23) and no interaction of life form and flower size (F_1,7_ = 2.13, *P* = 0.18).

In contrast to flower production (where small flowered species produce more flowers than larger flowered species), there was not a significant effect of flower size in any of the three most likely models analysing absolute values of maximum fruit production (Fig. 2c) or in the single most likely model of maximum fruit production scaled to tree size (Fig. 2d). Emergent species had a marginally greater value of maximum absolute fruit production than canopy species (Fig. 2c), and this term was significant in one of the three most likely models for this variable, but there was no difference between canopy and emergent species in maximum fruit production scaled to tree size (Fig. 2d). The nearest neighbour distance term was non-significant in all analyses of fruit production among species. The relationships between flower size and fruiting variables and pollination success (median number of pollen tubes per pollinated flower) were all supported by phylogenetically independent contrasts. Of the 12 species used in this study, only eight species exist in well resolved phylogenies [33], [34]. With the exception of *S. xanthophylla* all the *Shorea* species in our sample fall within the Red Meranti clade [34], with no apparent phylogenetic signal of calyx size.

### Pollination success

Analyses of the proportion of flowers pollinated identified five most likely models (Table S1c, Supplementary Information), but in all cases a positive effect of flower size was the only significant term (F_1,7_ = 55.97, *P*<0.001, Fig. 2e). Similarly, flower size was the only significant term in all the five most likely models analysing interspecific differences in the mean number of pollen tubes per pollinated flower (F_1,9_ = 45.72, *P*<0.0001, Fig. 2f). Differences in life form and in median nearest neighbour distance were not significant in these models of pollination success among species.

### Pollinator body size

**Figure 3:**
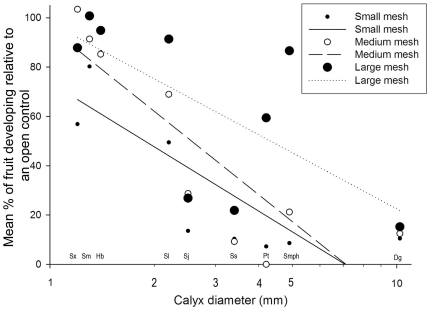
Effect of pollinator exclusion using bags with mesh apertures of 0.2 mm (solid line, small closed symbols), 2 mm (dashed line, open symbols) and 5 mm (dotted line, large closed symbols) on pollination success one month after anthesis. Pollination success is defined as the mean percentage of flowers that gave rise to immature fruit for 8 to 10 bags per treatment on 2 to 6 replicate trees per species, expressed as a percentage of the mean value for an equivalent sample of open-pollinated flowers on the same tree.

The experimental exclusion of pollinators based on body size resulted in a significant decline in pollination success with increasing flower size (*t* = −6.18, *P*<0.0001, Fig. 2). In small-flowered species (1.0–2.0 mm), such as *Shorea xanthophylla*, restricting access to pollinators had very little effect on the proportion of fruit produced relative to that expected among open pollinated flowers. In contrast, in the largest flowered species *Dipterocarpus grandiflorus* fruit set within treatments that limited pollinator access to small bodied insects was substantially reduced under all mesh sizes (Fig. 3 see Dg). The response to mesh bag treatments was significant (*t* = 2.76, *P* = 0.011), but the interaction of flower size and mesh bag treatment was not significant.

### Pollen dispersal distance and genetic relatedness between assigned parents

Paternity analysis revealed that short-distance mating events were far more common among the smaller flowered species *S. xanthophylla* (47% of matings within 50 m and 71% within 100 m of the mother tree) than the larger flowered species *P. tomentella* (13% within 50 m and 32% within 100 m) (χ^2^ = 17.96, *P*<0.001). The median (Wilcox rank sum  = 7325, *P*<0.0001) and mean (*t* = −4.609, *P*<0.0001) pollen dispersal distances for all assigned progeny showed a similar pattern: 63 m and 119 m in *S. xanthophylla* and 164 m and 177 m in *P. tomentella* respectively. Because the density of potential pollen donors within 100 m of mother trees was generally greater for *S. xanthophylla* than *P. tomentella*, we also tested for differences in pollen dispersal between the two species using a subset of mother trees that had equivalent distances to nearest flowering neighbours. In the subset of *S. xanthophylla* mothers, 32 percent of matings were assigned at less than 50 m and 34 percent at less than 100 m. The results of the TWOGENER analysis of pollen dispersal are summarised in Table 1. The differentiation in the pollen gene pool is significant in both species (*P*<0.01) and almost twice as great in *S. xanthophylla* than in *P. tomentella* (0.0624 and 0.0364 respectively). In this analysis the mean pollen dispersal distance for *S. xanthophylla* was estimated at 56 m (S.E. 2.88) and for *P. tomentella* 416 m (S.E. 29.93). The effective number of pollen donors per mother tree (N*_ep_*) was 8.01 in *S. xanthophylla* and 13.73 in *P. tomentella* (Table 1). The effective pollen neighbourhood area (*A_ep_*) ranged from 1.46 ha (*A_ep_^min^*) −14.62 ha (*A_ep_^max^*) in *S. xanthophylla* and from 8.43 ha (*A_ep_^min^*) −84.26 ha (*A_ep_^max^*) in *P. tomentella* (Table 1).

**Table 1:** Mean ± SEM calyx tube width (mm) for the 12 study species of Dipterocarpaceae sampled in Sepilok forest reserve (SFR).

					Number of sample trees (n)		
Species (code)	Life form †	Mean tree height	Calyx tube width (mm)	Median NN distance (m)	Flower & Fruit production ^a^	Pollination success ^b^	Pollinator Exclusion ^c^
*Shorea xanthophylla* (Sx)	Canopy	40 m (25 m) - 35 m*	1.2±0.1	19.7	29	26	3
*Shorea multiflora* (Sm)	Canopy	40 m (No estimate) - 35 m*	1.3±0.2	37.9	35	31	6
*Hopea beccariana* (Hb)	Canopy	45 m (35 m*)	1.4±0.1	200	14	11	6
*Shorea leprosula* (Sl)	Emergent	60 m (60 m) - 65 m*	2.2±0.2	85.1	55	33	6
*Shorea parvifolia* (Sp)	Emergent	65 m* (60 m)	2.3±0.1	69.7	6	6	0
*Shorea johorensis* (Sj)	Emergent	50 m (65 m*)	2.5±0.1	66.7	32	29	6
*Shorea macropteraa* (Smac)	Canopy	40*- 50 m (60 m)	3.0±0.2	118.2	6	6	0
*Shorea smithiana* (Ss)	Emergent	60 m* (55 m)	3.4±0.2	91.8	22	10	3
*Shorea beccariana* (Sb)	Emergent	60 m (55 m*)	3.6±0.3	39.5	53	38	0
*Parashorea tomentella* (Pt)	Emergent	65 m (65 m*)	4.2±0.1	50.5	29	25	6
*Shorea macrophylla* (Smph)	Emergent	45 m (45 m)	4.9±0.2	N/A	N/A	N/A	2
*Dipterocarpus grandiflorus* (Dg)	Emergent	45 m * (45 m)	10.2±0.7	24.8	30	18	3

Mean tree heights are from Ashton (2004), except values in brackets which are from [91] and those marked *, which were measured directly at SFR or obtained from a published source equivalent to our own measure. Median two nearest neighbouring (NN) distance to flowering conspecific trees; number of sample trees (n) for a) quantification of flower and fruit production; b) pollination success, based on pollen tube analysis and c) trees for experimental pollinator exclusion.

† Following Ashton [89].

Genetic relatedness between mothers and assigned fathers was an order of magnitude higher in progeny of the small-flowered species *S. xanthophylla* than in the larger flowered species *P. tomentella* (mean pairwise kinship coefficient  = 0.033 (S.E. 0.008) and 0.002 (S.E. 0.01) respectively; *t* = 2.195, df = 229, *P* = 0.028). The mating system parameters based upon the progeny arrays indicate that both species are extensively outcrossing (*t_m_* = 0.996 and *t_m_* = 0.907 in *S. xanthophylla* and *P. tomentella* respectively). However, the parental inbreeding coefficient and biparental inbreeding were both greater in *S. xanthophylla* (see supporting information Table S2).

## Discussion

### Flower size/number trade-off and fecundity

We confirmed that flower size scaled negatively with flower number across 11 dipterocarp species. Although canopy species produced fewer flowers for their flower size than predicted by the scaling relationship for emergent species, all species fit the same negative relationship when flower production was scaled to tree size. These relationships suggest that the trade-off between flower size and potential flower production was consistent across all species, but subject to allometric constraints. These observations are robust to phylogenetic comparison These results are consistent with other studies that suggest that a flower size vs number trade-off is common in angiosperms [7], . Maximum fruit production per tree, in contrast to flower production, did not scale negatively with flower size. This indicates that the potential fecundity offered by a large number of small flowers was offset by the reduced likelihood that each of these flowers would be pollinated and develop into a mature fruit. Our results suggest that processes operating between flower production and fruit dispersal contribute to equalising fecundity among dipterocarp species. Below we interpret our findings and discuss the range of alternative hypotheses that could lead to the observed patterns of fecundity.

Fruit production in larger fruited species could be resource limited, leading to larger fruits being produced in fewer numbers [11]. However, we exclude this hypothesis for two reasons. First, the scaling relationships were determined for trees representing the upper quartile for flower production, which are least likely to be resource-limited among the sample. Secondly, fruit size scales positively with flower size in dipterocarps (C.R. Maycock *et al.*, unpubl. data), therefore small-flowered species would have lower overall per capita fruit mass, even though fruit number remains the same, a scenario that is at odds with the expectations of resource limited fruit production.

Experimental exclusion of all potential pollinators except thrips reduced pollination success in large-flowered but not small-flowered species. This result suggests that the role of thrips in pollination is important for small-flowered species of dipterocarp such as *Shorea xanthophylla* but less so as flower size increases. Thrips are abundant in the flowers of all the dipterocarps examined in this study and are widespread in the flowers of many non-dipterocarps in the same forest (C.R. Maycock, *et al*. unpubl. data). Hence their abundance is unlikely to limit pollination success in the small-flowered species, and therefore we reject pollinator limitation as a determinant of the flower size-dependent reduction in pollination success.

There are several concurring lines of evidence to support the hypothesis that not only do large-flowered species receive a higher proportion of pollen (as empirically demonstrated here for 11 species) but also that pollen received is of greater genetic quality (*i.e.*, more compatible). First, our examination of pollen tube growth among species showed that both the proportion of pollinated flowers and mean number of pollen tubes per pollinated flower was greater in larger-flowered species. This implies that either smaller-flowered species receive fewer per capita visits by pollinators than larger-flowered species, and/or that a higher proportion of pollination events result in the transfer of non-compatible self-pollen that is blocked at the stigmatic surface or at the early stages of pollen tube growth. Dipterocarp species are considered to be self-incompatible [28], and although there is evidence that selfing can lead to fruit production, these fruit are preferentially aborted at an early stage of maturation [36], [37]. This behaviour may have evolved as a predator avoidance strategy [38].

Second, the pollinator exclusion experiment also revealed that fruit set increased as access to larger pollinators was permitted by larger mesh sizes in larger flowered species. The exception to this pattern was the largest flowered species, *Dipterocarpus grandiflorus*, which had significantly reduced fruit set even with the largest mesh (5 mm). This outcome suggests that the importance of larger pollinators increases as flower size increases and, for the largest flowered species, *D. grandiflorus*, pollinators that exceed 5 mm in size, such as giant honey bees *Apis dorsata*, large scarabid beetles and Sphingidae and Noctuidae moths, play an important role. We conclude that the role of small-bodied insects in dipterocarp pollination, in particular thrips, declines with increasing flower size. The relevance of this finding in the context of putative trade-offs is that the larger bodied pollinators may transfer pollen over greater distances and may therefore be more effective agents for outcrossing [13], [39].

#### Differential pollen dispersal

Our study provides evidence that larger bodied pollinators disperse pollen over greater distances than smaller bodied pollinators in dipterocarps. Our paternity analysis and indirect estimates of pollen dispersal demonstrated that short-distance pollen dispersal events were more frequent, and that the mean pollen dispersal distance were shorter, for the small-flowered species *Shorea xanthophylla* than for *Parashorea tomentella*, independent of density of flowering conspecifics. Progeny collected from a subset of *S. xanthophylla* mother trees with an equivalent density of flowering conspecifics to that of *P. tomentella* were assigned more frequently to nearer pollen donors (<100 m) than for *P. tomentella*. Other studies have estimated mean pollen dispersal distances in dipterocarp species to lie in the range 175–207 m [22], [25], [26]. For the five dipterocarp species where pollen dispersal distances have been quantified, representing flower sizes ranging over two orders of magnitude, mean pollen dispersal distance increases with flower size, with the lowest mean dispersal distances in *S. xanthophylla* and greatest dispersal distances in *Dipterocarpus tempehes* (Table 2). Such a pattern remains inconclusive, as it is based on only five species, and other factors may influence pollen-mediated gene flow, including differences in the density of flowering conspecifics. Nevertheless, the results are suggestive that increasing flower size corresponds to higher pollen flow distances, which we interpret as being mediated by the larger-bodied pollinators attracted by the larger flowers. Hence, all else being equal, these larger-flowered species experience higher outcrossing rates and are less susceptible to inbreeding. This corresponds to a higher quality of pollination service for large flowered species, which compensates for the relatively few flowers produced by these species.

**Table 2:** Summary table of pollen dispersal from TWOGENER analysis in two species of dipterocarp with contrasting flower size.

Species	d/trees ha	Φft	δ (m)	*N_ep_*	*A_ep_^min^* (ha)	*A_ep_^max^* (ha)
*S. xanthophylla*	5.48	0.0624**	56.27 (43.08–69.48)	8.01	1.46	14.62
*P. tomentella*	1.63	0.0364**	416.25 (292.84–539.67)	13.73	8.43	84.26

Density of adult trees (d); differentiation in pollen gene pool (Φ*_ft_*); mean pollen dispersal distance(*δ*); effective number of pollen donors (*N_ep_*); minimum effective pollen neighbourhood area (*A_ep_^min^*) and maximum effective pollen neighbourhood area (*A_ep_^max^*) following Smouse *et al* 2001.

** Significant at *P*<0.01, values in parentheses are 95% confidence intervals.

Inbreeding and thus reduced fecundity through inbreeding depression are predicted to be greater in species where related individuals are highly aggregated. Seed dispersal limitation and habitat specialisation are common in dipterocarps [30], [31], and both processes can lead to aggregation of related individuals [40], [41], [42]. This is measurable as fine-scale spatial genetic structure (SGS) within populations. A strong SGS signal would not be expected if gene flow by pollen or seed dispersal is extensive. Dipterocarp seed dispersal is known to occur over short distances [43], [44], [45], [46], [47], which suggests that long distance pollen flow is likely to be the primary agent undermining SGS. The evidence for fine-scale SGS observed in comparable studies of dipterocarp species [11], [26], [29], [32], [48], [49] suggests that SGS is common in the Dipterocarpaceae, but weaker in larger flowered species. This interpretation is consistent with our pollinator exclusion experiments, and other studies [25], [50], [51] that identify the importance of large insects such as *Apis dorsata* and sphingid moths for pollination of large-flowered dipterocarps.

Significant fine-scale SGS combined with limited pollen dispersal may impose a fitness cost due to elevated inbreeding depression or incompatible matings in self-incompatible species (Fig. 1), and this is likely to be more substantial among smaller flowered species for the reasons given above. Such processes would lead to a genetic cost under limited pollen dispersal and is supported by evidence of a higher frequency of matings between more closely-related individuals in the smaller-flowered species *Shorea xanthophylla* than in *Parashorea tomentella*. High neighbourhood genetic relatedness has been shown to interact with density to increase the proportion of aborted fruit in the insect pollinated tropical tree *Jacaranda copaia* [41], which supports our hypothesis that genetic processes contribute to the trade-off between flower size and pollination success (Fig. 1).

#### Differential Fecundity

As a component of plant fitness, fecundity is represented in many models of population persistence and species coexistence [52], [53], [54], [55]. However, empirical investigations of coexistence in tropical trees have focused more on plant responses to abiotic resource availability [56], [57], [58]. These studies do not support an unequivocal role for habitat partitioning in the maintenance of the diverse tropical tree communities observed in Borneo. While our study is limited to only a few species, it does highlight the potential importance of partitioning of the pollinator niche as an important biotic resource, and also provides the basis for postulating a series of trade-offs (depicted in Figure 1) linking fruit production to flower size, flower number and pollination systems within the Dipterocarpaceae. These mechanisms may well contribute to equalisation of fecundity among species, and warrant further investigation as a novel mechanism for promoting coexistence.

### Conservation implications

Our findings have implications for the conservation of genetic and species level diversity. First, our results highlight the potential for differential vulnerability of species to inbreeding, genetic drift and pollen limitation as a result of habitat fragmentation [15], [59], [60]. Larger-flowered dipterocarps may be less vulnerable to fragmentation as larger-bodied pollinators have a greater capacity to disperse between forest fragments [15], [61], [62] and thus maintain larger effective populations of forest trees. Second, the genetic processes invoked here also have clear implications for tropical forest restoration, and specifically the collection of seeds for generating planting stock. The genetic diversity of seed collections from small flowered species may be more vulnerable to cryptic processes, such as an elevated proportion of inbred progeny, which may compromise the long-term viability of forest restoration [63]. Hence we emphasise the importance of maintaining the integrity of pollinator communities and dispersal corridors between forest fragments for tropical forest conservation.

### Conclusions

Our study suggests that a trade-off exists between flower size and number that, coupled with a positive scaling between flower size and pollination success, reduces the variance in per tree fruit production between species. We have shown that species with small flowers produce them in greater numbers, but these flowers are pollinated by small-bodied insects that are less likely to disperse pollen over long distances. This gives rise to limited gene flow by pollination and, coupled with adult populations with strong spatial genetic structuring, increases the likelihood of mating between related individuals in small-flowered species. As mean flower size increases across species, flower number per tree declines, but the likelihood of receiving pollen from unrelated (more distant) conspecifics increases because larger-bodied pollinators such as sphingid moths and *Apis dorsata* become increasingly important as pollinators. Thus a trade-off embedded in the relationship between flower size and the pollination ecology of co-occurring tropical trees contributes to reducing variance in per capita fecundity among species and life-forms. Although we cannot infer that fecundity relates directly to life-time mean fitness, seed production is an important component of plant reproductive success. Inbreeding has been shown to have direct implications on differential seed mass in dipterocarps [64] and germination rates and survival in other tropical tree species [65], [66], [67]. It is therefore plausible that these processes reduce fitness inequalities among species and thereby delay competitive exclusion. This perspective suggests that partitioning of pollinators may contribute to maintaining the diversity of species in this ecologically and economically important family of tropical trees, and that the pollination component of the regeneration niche may play a more substantial role in maintaining species diversity than has been recognised previously.

## Materials and Methods

### Study site and species

The study site was the Sepilok Forest Reserve (SFR: 5°47′–5°52′ N, 117°55′–118°03′ E), which is about 24 km west of the state capital of Sandakan on the east coast of Sabah, Malaysia (SI Fig. S1). SFR is a “Class VI virgin jungle reserve” gazetted in 1930, managed by the Forest Research Centre Sabah for forest protection and research. SFR supports lowland dipterocarp and heath forest (described by Fox [68]). Mean annual temperature falls in the range of 26.7–27.7°C and mean annual rainfall is 2929±134 mm (Malaysian Meteorological Service, unpubl. data).

### Flower size, flower number and fruit production

We examined the trade-offs among flower size and total flower and fruit production across 12 dipterocarp species during two minor flowering events between July 2001 and November 2002 supplemented by information obtained during flowering events in 2006–2008 (Table 3). Mean flower size of these species, measured as calyx tube diameter on an average sample of 700 flowers per species from 6 to 55 individuals, varied in the range 1.2 mm–10.2 mm (Table 3). Flowering trees were located using binoculars along an extensive trail system, in an area of approximately 640 ha. A mean of 22 trees were sampled for each species (Table 3). Diameter at breast height (dbh) and crown width were measured for each tree, and their positions were recorded using a GPS (Magellen 315, Thales Navigation, Santa Clara, USA). Similarly, the nearest two flowering neighbours for each tree sampled were located and their positions recorded as above. Phylogenetically independent contrasts (PICs) were used to examine trade-offs between flower size and total flower production, pollination success, the mean number of pollen tubes per pollinated flower or maximum total fruit production to take account of phylogenetic inertia within closely related species [69]. We applied the relationships determined from a recent dipterocarp phylogeny using *PgiC* gene sequence [34] which includes seven of the 12 study species. The PIC analysis was conducted using the computer program Compare 4.6b on the transformed variables [70]. Following PIC analyses, the Y-variable contrasts were regressed on the X-variable contrasts as outlined in [71].

**Table 3:** Summary statistics of pollen dispersal from paternity analysis in dipterocarp species with a range of flower size, including the number of genotyped offspring (n); progeny type used in the paternity analysis: mature embryos directly collected from mother (ME); forest floor seedlings (FS), or Saplings (Sp); seedlings germinated from seeds collected below putative mothers (GS); proportion of total embryos assigned at 80% confidence level (% *asgn* 80%); mean pollen dispersal distance across all assigned embryos (*MPDd*), and sample area over which paternity analysis was conducted (PN area); N/A  =  not available.

Species	Calyx tubeWidth (mm)	n	Progeny type	% *asgn* 80%	*MPDd* (m)	S.E	PN area (ha)	source
*S. xanthophylla*	1.2	456	ME	68	119	8.4	100	This study
*S. lumutensis*	2.2	182	GS	63	175	N/A	8	[26]
*P. tomentella*	4.2	408	ME	26	176	14.67	100	This study
*N. heimii*	10.0	248	FS & Sp	37	191	N/A	42	[22]
*D. tempehes*	10.0	335	FS	88	207	N/A	70	[25]

Four 1 m^2^ mesh traps raised 1 m above the ground were installed under each tree to estimate flower production. The traps were placed approximately 5 m from the trunk, and at 90° intervals from a random azimuth. All traps were installed prior to the commencement of flower fall and their contents were collected approximately every three days until the end of fruit-fall. The samples were air dried and sorted into fractions representing buds, flowers and fruit, and the fractions were counted and weighed. Flower counts were based on calyxes, which we presume to be subjected to less lateral dispersal by wind than corollas. Total flower production was estimated by multiplying the total number of buds, flowers and fruit captured by the traps (expressed per m^2^) by the projected area of the tree crown. The flowering events we sampled for this paper were not general flowering events sensu [72], and a high proportion of flowering individuals produced only a small number of flowers on relatively few branches. Such flowering events are thought to contribute very little to population-level fecundity [73]. Therefore we estimated mean maximum flower and fruit production from the upper quartile of values per species (n = 2–14 individuals per species). Because basal area alone is insufficient to account for differences in architecture and allometry among species and to account for effects of canopy exposure on flower production, we present values of maximum flower production scaled to tree size:
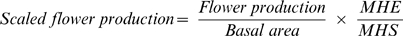
where MHE/MSE is a scaling factor for height calculated by dividing the maximum height of emergents at the study site (MHE: 65 m) by the maximum height of the study species (MHS). Basal area was determined by direct measurement for each tree sampled. Maximum height of the emergent trees in the SFR and maximum height of each species were estimated to the nearest 5 m using a measuring line dropped from the canopy. Total fruit production was estimated from the mean number of fruit captured per trap multiplied by the projected area of the tree crown.

### Pollination success

We examined pollen tube growth as a measure of pollination success in 11 of the same 12 dipterocarp species using flower samples collected during 2001–2002 (*H. beccariana*, *S. beccariana*, *S. johorensis*, *S. leprosula*, *S. macroptera*, *S. multiflora*, *S. parvifolia*, *S. smithiana* and *S. xanthophylla*,) or 2006–2008 (*D. grandiflorus* and *P tomentella*) (Table 3). Flower samples were obtained using mixed rope-climbing techniques to gain access to the canopy. Inflorescences were collected directly from each tree at five randomly selected locations within the crown. Flowers that had recently shed their corollas were removed from the inflorescences. We collected a minimum of 500 flowers from each tree for the *Shorea* spp and *H. beccariana*, while the low flower densities of *D. grandiflorus* and *P. tomentella* limited the sample to approximately 50 and 100 flowers per tree, respectively. All flowers were stored in formalin-acetic-alcohol (FAA). Mean calyx diameters for each tree were determined from direct measurement on 25 randomly selected flowers. The styles of the flowers were stained with 1 percent aniline blue solution and the number of pollen tubes in each were determined using fluorescence microscopy as outlined in Ghazoul *et al.*, [21]. We examined pollen tubes within 25200 flowers sampled randomly from the flower collections taken from a total of 638 individuals of the 11 species.

### Pollinator body size

Mesh bags were used to exclude visitors from inflorescences prior to anthesis on 2–6 trees from each of nine of the 12 study species that represented an order-of-magnitude range in flower size and flowered between May 2006 and May 2008 (Table 1). Ten replicate inflorescences per tree were used for each of the following treatments: (a) open pollination (no mesh); (b) exclusion of large pollinators (5 mm mesh); (c) exclusion of medium-sized and large pollinators (2 mm mesh); and (d) exclusion of all pollinators except thrips (0.2 mm mesh). Control large mesh bags containing fly trap paper confirmed that large mesh bags did not inhibit visitation by smaller pollinators. The bags were removed at the end of anthesis. Fruit initiation and development were monitored every five days throughout the first month, and then every 10 days until fruit maturity. The effect of mesh bag treatments is expressed as percent pollination success relative to the open pollination treatment one month after anthesis.

### Pollen dispersal distances

Pollen dispersal distance was estimated using paternity analysis based on samples of mature fruit collected from known mother trees of *Shorea xanthophylla* (mean calyx diameter 1.2 mm) and *Parashorea tomentella* (mean calyx diameter 4.6 mm). These species were selected because this contrast in flower size was predicted to generate differences in pollen dispersal distance based on our sampling of their flower visitor communities (C.R. Maycock *et al.*, unpubl. data), and because their densities of flowering individuals maximised sampling effort for the available resources. We applied identical sampling strategies in both species, combining the genotypes of mother and progeny with genotypes from all potential pollen donors in mapped populations within 500 m of each mother trees. Because flowering tree density can have a high variance, both within and among species, our sampling design enables us to explicitly control for this.

#### DNA sampling, extraction and genotyping

All sampling was carried out between April 2006 and September 2008. 20–35 mature fruits were sampled from each of 20 and 17 mother trees of *Shorea xanthophylla* and *Parashorea tomentella* respectively. Fresh fruits were dissected and embryos removed and dried in Sigma™ silica gel prior to DNA extraction. In addition to sampling inner bark from each mother tree, inner bark was sampled from all flowering con-specifics within a 500 m radius of each mother and the location of all adult trees recorded with a GPS. For adult trees, fresh inner bark (cambium) was collected using a 2 cm diameter leather punch and a hammer following Colpaert *et al.* [74]. The fresh samples were placed in labelled tea bags and dried in Sigma™ silica gel within 12 hours of sampling, and stored under the same conditions prior to DNA extraction. Total genomic DNA was extracted from approx. 0.03 g of silica dried material (inner bark, embryo or leaf, depending on sample type) and ground to a fine powder using a Qiagen Mixer-mill™ with Retsch © stainless steel ‘cone balls’ and the Qiagen DNAeasy™ 96-well-plate plant extraction system. For the paternity analysis the genotype of each mother tree, candidate father and embryo was determined at nine polymorphic, genomic, nuclear microsatellite loci. Primers are summarised in Supplementary Information Table S3. The forward primer of each locus was 5- prime end labelled with one of four florescent-labelled dyes (FAM, NED, PET or VIC) to enable multiplexing of multiple loci during fragment analysis. All PCR products were quantified using an ABI 3730xl DNA Analyser (Applied Biosystems) and sample genotypes scored using Genemapper v.4.0™ software (Applied Biosystems).

To establish whether data from different microsatellite loci were independent, genotypic linkage disequilibrium was tested between all pairs of loci within each species adult sample using GENEPOP 4.0 [75] with the following settings; dememorization: 1000, batch: 100, iteration per batch: 5000. No evidence for significant linkage disequilibrium was detected.

#### Direct estimates of pollen dispersal

Mother trees were sampled based upon the abundance of fruits and accessibility. Using multilocus genotypes (9 loci, see Table S3 Supporting information) of progeny and known mother trees we applied a maximum likelihood exclusion analysis in CERVUS 3.0, to exclude candidate fathers [76], [77]. Candidate fathers were defined as all flowering conspecifics within a 500 m radius of each mother tree. Pollen dispersal distance was deduced for each embryo based on the position of the mother tree and the assigned pollen parent (father) within the 500 m radius. Simulations of paternity were run using the allele frequencies of all adult reproductive trees and the following settings: 10000 cycles; minimum number of loci typed 5, mothers as known parents, test for selfing (*i.e.*, mothers also included as candidate fathers); the flowering conspecifics within 500 m of each mother were set as the candidate fathers for progeny from that mother only; 1 percent for proportion of loci mistyped, and 87 percent for proportion of loci genotyped. The proportion of candidate fathers sampled was set at 80 percent, which was considered a conservative estimate based upon the observed frequency of flowering conspecifics across the entire SFR. Assignment was based upon the 80% confidence level of the critical LOD score.

Based upon paternity assignment, 68 percent of progeny (310 of 456) from 20 mothers trees of *S. xanthophylla* and 26 percent of progeny (106 of 408) from 17 mother trees of *P. tomentella* could be assigned with an 80% confidence within 500 m of the mother tree. For all progeny that could not be assigned at this level of confidence the pollen donors were assumed to be beyond a 500 m radius of each mother tree. This assumption would be supported by evidence that the gene pool of the genotyped progeny differed significantly from that of the pollen donor pool within the 500 m radius. To test this we examined the frequency of rare alleles (abundance <0.01) in the embryo cohorts of each species. In *P. tomentella* 92 percent of the rare alleles (<0.01) are observed only in the embryos, not in the mothers or neighbouring adults, thus must have come from outside the 500 m radius we sampled. In contrast in *S. xanthophylla* only 49 percent of rare alleles were unique to the embryo cohort.

#### Indirect estimate of pollen dispersal

Pollen dispersal was also evaluated using the TWOGENER [78], [79] approach with POLDISP [80]. TWOGENER estimates the heterogeneity of the pollen gene pool sampled within a set of maternal trees (Φ *_FT_*) using AMOVA [81]. This has the added advantage that it provides an estimate of contemporary gene flow over the scale of the landscape which is less sensitive to flowering tree density and complementary to the more localised estimate of the paternity analysis. The mean effective pollen dispersal distance (δ) was calculated assuming the following dispersal models; normal, bivariate normal and anisotropic normal, following [79], [82], [83] in *S. xanthophylla* and *P. tomentella*. The density of adult trees was calculated using our detailed spatial distribution data of our focal species within SFR (Table 1). The effective number of pollen donors *N_ep_* and effective pollination neighbourhood area (*Â_ep_*) were calculated as follows: *N_ep_* = *1/2Φ_ft_* and *Â_e_ = N_ep_*/*d_e_* where *d_e_* is the effective density of trees. We used a range of values of *d_e_* including those derived from the TWOGENER analysis. Because the effective density of flowering adults may vary markedly from year to year we calculated *N_ep_* assuming a range of densities based upon the census density of reproductive adults (*d*) and *d*/2, *d*/4 and *d*/10 following the approach of [84] after [79].

### Inbreeding of progeny

The relatedness between mothers and putative fathers, derived from the paternity analysis, was estimated for each of the assigned progeny using SPAGEDI 1.2 g [85]. Here we calculated the individual and mean kinship coefficient *F* [86] for each parent pair assigned by CERVUS [76] within *S. xanthophylla* and *P. tomentella* (n = 229 and 106 respectively). Mean kinship coefficients *F* have been used to express the relatedness between mating individuals in ants [87] and are useful as they are directly comparable to measures of spatial genetic structure among species. We supplement the measures of relatedness between mating pairs with estimates of the number of pollen donors per seed crop (Nep) and multilocus outcrossing estimates using the program MLTR v 2.4 [88].

### Statistical analysis

Linear models were used to investigate trade-offs among species' between log-transformed flower size and: (1) log-transformed maximum flower production; (2) arcsine-transformed percentage of flowers pollinated; (3) log-transformed mean number of pollen tubes per pollinated flower; and (4) log-transformed maximum fruit production. We included life-form (canopy or emergent following Ashton [89]), as an additional independent variable in all models. For analyses of pollination success and fruit production (2–4) we also included median values of the mean distance to the two nearest flowering trees (for pollination success) or fruiting trees (for fruit production) as an independent variable to account for possible effects of differences between species in the density of reproductive trees. We compared models containing all possible combinations of one or more explanatory variables and their interactions and used AIC values to select the most likely models. Models with AIC values that differed by less than two were judged equally valid [90] but F statistics and probability values reported in this paper are taken from the model with the lowest AIC value.

The effects of pollinator exclusion on pollination success were analysed using a generalised linear model with log-transformed calyx diameter and mesh bag treatment as fixed explanatory variables. The non-significant interaction term was removed from the model. Pollination success was expressed as the proportion of fruits within each treatment surviving to one month after the end of anthesis, relative to open pollination.

To test the null hypothesis of no difference in the frequency of short distance mating we conducted a chi-squared test on counts of pollen dispersal within distance classes <50 m, 50–99.9 m, 100–199.9 m and 200–500 m in *S. xanthophylla* and *P. tomentella.* Average pollen dispersal distances over all assigned embryos were compared between *S. xanthophylla* and *P. tomentella* using a Student *t*-test (arithmetic mean) and Wilcox test (median). To determine the significance of inbreeding of progeny as a function of flower size we used a Student *t*-test to test the null hypothesis of no difference in mean kinship coefficient. All statistics were conducted using R (R Development Core Team 2009).

## Supporting Information

Figure S1 Location of Sepilok Forest Reserve, boundary demarked by orange line together with distribution of flowering trees of *Parashorea tomentella* and *Shorea xanthophylla* used for paternity analysis in. The two grey ellipses indicate the subset of mother trees from *S. xanthophylla* which had comparable local density of flowering conspecifics to *P. tomentella*.
(DOC)

Table S1 (**a**) AIC and Δ_AIC_ values from four candidate models of log-transformed absolute (unscaled) and scaled flower production for 11 dipterocarp species at Sepilok Forest Reserve, Sabah. The most likely models are shown in bold. (**b**) AIC and Δ_AIC_ values from 18 candidate models of arcsine square root – transformed proportion of flowers pollinated and log-transformed mean number of pollen tubes per pollinated flower for 11 dipterocarp species at Sepilok Forest Reserve, Sabah. The most likely models are shown in bold. logFS, log-transformed flower size; LF, life-form; NND, median values of the mean distance to the two nearest flowering trees. (**c**) AIC and Δ_AIC_ values from 18 candidate models of log-transformed absolute (unscaled) and scaled fruit production for 11 dipterocarp species at Sepilok Forest Reserve, Sabah. The most likely models are shown in bold. logFS, log-transformed flower size; LF, life-form; NND, median values of the mean distance to the two nearest flowering trees.
(DOCX)

Table S2 Mating system statistics for progeny of *Shorea xanthophylla* and *Parashorea tomentella* based upon 9 microsatellites loci. Number of progeny genotypes (*N*); multilocus outcrossing rate (*t_m_*); single locus outcrossing rate (*t_s_*); biparental inbreeding as defined by the difference between multilocus and single locus outcrossing rates (*t_m_ - t_s_*); Parental inbreeding coefficient. Values in parentheses are standard error (SE) based upon 100 bootstraps.
(DOCX)

Table S3 Summary of the 11 microsatellite primers used for paternity analysis and quantification of relatedness between assigned parents in two dipterocarp species *Shorea xanthophylla* and *Parashorea tomentella*. Number of alleles (Na); observed heterozygosity (*H_obs_*); expected heterozygosity (*H*_e_); paternity non-exclusion probability at each locus (*N-P_E_*) and total exclusion probability over all loci (*P_E_*) given known mother. *^a^* Redesigned primers based on published primers. *^b^* Newly developed microsatellite primers. ^c^ Published primers.
(DOCX)
