# Supplementary material for: Ecological Implications of a Flower Size/Number Trade-Off in Tropical Forest Trees
Source: PLoS One. 2011 Feb 1;6(2):e16111. doi: 10.1371/journal.pone.0016111 (PMC3052255; doi:10.1371/journal.pone.0016111)
Supplement: Table S1 — (a) AIC and ΔAIC values from four candidate models of log-transformed absolute (unscaled) and scaled flower production for 11 dipterocarp species at Sepilok Forest Reserve, Sabah. The most likely models are shown in bold. (b) AIC and ΔAIC values from 18 candidate models of arcsine square root – transformed proportion of flowers pollinated and log-transformed mean number of pollen tubes per pollinated flower for 11 dipterocarp species at Sepilok Forest Reserve, Sabah. The most likely models are shown in bold. logFS, log-transformed flower size; LF, life-form; NND, median values of the mean distance to the two nearest flowering trees. (c) AIC and ΔAIC values from 18 candidate models of log-transformed absolute (unscaled) and scaled fruit production for 11 dipterocarp species at Sepilok Forest Reserve, Sabah. The most likely models are shown in bold. logFS, log-transformed flower size; LF, life-form; NND, median values of the mean distance to the two nearest flowering trees. (DOCX) [file pone.0016111.s002.docx]

**SUPPORTING INFORMATION**

Additional supporting information may be found in the online version of this article:

**The Flower size/number trade-off and its ecological consequences for tropical forest trees of Borneo.**

Table S1a. AIC and ∆_AIC_ values from four candidate models of log-transformed absolute (unscaled) and scaled flower production for 11 dipterocarp species at Sepilok Forest Reserve, Sabah. The most likely models are shown in bold.

|  | Log unscaled flower production | | Log scaled flower production | |
| --- | --- | --- | --- | --- |
| Candidate models | AIC | ∆_AIC_ | AIC | ∆_AIC_ |
| *Single factor models* |  |  |  |  |
| log (flower size) | 17.11 | 23.57 | **-4.22** | **0.82** |
| Life form | 20.62 | 27.08 | 14.93 | 19.97 |
| *Two factor model* |  |  |  |  |
| log (flower size) + Life form | 7.10 | 13.56 | **-4.11** | **0.93** |
| *Two factor model with interaction* |  |  |  |  |
| log (flower size) + Life form + log (flower size)*Life form | **-6.46** | **0.00** | **-5.04** | **0.00** |

Table S1b. AIC and ∆_AIC_ values from 18 candidate models of arcsine square root – transformed proportion of flowers pollinated and log-transformed mean number of pollen tubes per pollinated flower for 11 dipterocarp species at Sepilok Forest Reserve, Sabah. The most likely models are shown in bold. logFS, log-transformed flower size; LF, life-form; NND, median values of the mean distance to the two nearest flowering trees.

|  | Arcsin sqrt pollination success | | Log pollen tube number | |
| --- | --- | --- | --- | --- |
| Candidate models | AIC | ∆_AIC_ | AIC | ∆_AIC_ |
| *Single factor models* |  |  |  |  |
| log (flower size) [logFS] | **-8.06** | **1.09** | **-12.14** | **0.00** |
| Life form [LF] | 4.74 | 13.89 | 2.35 | 14.49 |
| Nearest neighbour distance [NND] | 10.94 | 20.09 | 5.91 | 18.05 |
| *Two factor models* |  |  |  |  |
| log (flower size) + Life form | **-7.18** | **1.97** | **-10.26** | **1.88** |
| log (flower size) + Nearest neighbour distance | -6.06 | 3.09 | **-11.43** | **0.71** |
| Life form + Nearest neighbour distance | 6.69 | 15.84 | 3.60 | 15.74 |
| *Three factor model* |  |  |  |  |
| log (flower size) + Life form + Nearest neighbour distance | -5.22 | 3.93 | -9.45 | 2.69 |
| *Two factor models with interaction* |  |  |  |  |
| log (flower size) + Life form + log (flower size)*Life form | -6.68 | 2.47 | **-11.37** | **0.77** |
| logFS + Nearest neighbour distance + logFS* Nearest neighbour distance | **-9.15** | **0.00** | **-10.38** | **1.76** |
| Life form + Nearest neighbour distance + Life form* Nearest neighbour distance | 6.83 | 15.98 | 0.41 | 12.55 |
| *Three factor models with one interaction* |  |  |  |  |
| logFS + NND + LF + logFS*LF | -4.80 | 4.35 | -9.61 | 2.53 |
| logFS + NND + LF + logFS*NND | **-8.43** | **0.72** | -8.44 | 3.70 |
| logFS + NND + LF + LF*NND | -5.91 | 3.24 | -7.51 | 4.63 |
| *Three factor models with two interactions* |  |  |  |  |
| logFS + NND + LF + logFS*LF + logFS*NND | **-7.48** | **1.67** | -7.73 | 4.41 |
| logFS + NND + LF + logFS*LF + LF*NND | -4.46 | 4.69 | -7.77 | 4.37 |
| logFS + NND + LF + logFS*NND + LF*NND | -6.43 | 2.72 | -7.18 | 4.96 |
| *Three factor model with three interactions* |  |  |  |  |
| logFS + NND + LF + logFS*LF + logFS*NND + LF*NND | -6.31 | 2.84 | -5.77 | 6.37 |
| *Three factor model with all interactions* |  |  |  |  |
| logFS + NND + LF + logFS*LF + logFS*NND + LF*NND + logFS*NND*LF | -4.85 | 4.30 | -5.05 | 7.09 |

Table S1c.AIC and ∆_AIC_ values from 18 candidate models of log-transformed absolute (unscaled) and scaled fruit production for 11 dipterocarp species at Sepilok Forest Reserve, Sabah. The most likely models are shown in bold. logFS, log-transformed flower size; LF, life-form; NND, median values of the mean distance to the two nearest flowering trees.

|  | Log unscaled fruit production | | Log scaled fruit production | |
| --- | --- | --- | --- | --- |
| Candidate models | AIC | ∆_AIC_ | AIC | ∆_AIC_ |
| *Single factor models* |  |  |  |  |
| log (flower size) [logFS] | 13.63 | 5.71 | 11.88 | 8.81 |
| Life form [LF] | 10.34 | 2.42 | 11.83 | 8.76 |
| Nearest neighbour distance [NND] | 12.54 | 4.62 | 10.42 | 7.35 |
| *Two factor models* |  |  |  |  |
| log (flower size) + Life form | **9.23** | **1.31** | 13.43 | 10.36 |
| log (flower size) + Nearest neighbour distance | 14.53 | 6.61 | 9.47 | 6.40 |
| Life form + Nearest neighbour distance | 11.91 | 3.99 | 11.44 | 8.37 |
| *Three factor model* |  |  |  |  |
| log (flower size) + Life form + Nearest neighbour distance | 11.22 | 3.30 | 11.41 | 8.34 |
| *Two factor models with interaction* |  |  |  |  |
| log (flower size) + Life form + log (flower size)*Life form | 11.23 | 3.31 | 12.52 | 9.45 |
| logFS + Nearest neighbour distance + logFS* Nearest neighbour distance | 13.22 | 5.30 | 8.65 | 5.58 |
| Life form + Nearest neighbour distance + Life form* Nearest neighbour distance | 12.91 | 4.99 | 11.76 | 8.69 |
| *Three factor models with one interaction* |  |  |  |  |
| logFS + NND + LF + logFS*LF | 13.22 | 5.30 | 12.89 | 9.82 |
| logFS + NND + LF + logFS*NND | 11.51 | 3.59 | 10.47 | 7.40 |
| logFS + NND + LF + LF*NND | 13.21 | 5.29 | 13.19 | 10.12 |
| *Three factor models with two interactions* |  |  |  |  |
| logFS + NND + LF + logFS*LF + logFS*NND | 11.48 | 3.56 | 11.80 | 8.73 |
| logFS + NND + LF + logFS*LF + LF*NND | 11.16 | 3.24 | 9.40 | 6.33 |
| logFS + NND + LF + logFS*NND + LF*NND | 10.57 | 2.65 | 11.16 | 8.09 |
| *Three factor model with three interactions* |  |  |  |  |
| logFS + NND + LF + logFS*LF + logFS*NND + LF*NND | **9.81** | **1.89** | 8.64 | 5.57 |
| *Three factor model with all interactions* |  |  |  |  |
| logFS + NND + LF + logFS*LF + logFS*NND + LF*NND + logFS*NND*LF | **7.92** | **0.00** | **3.07** | **0.00** |
