# Supplementary material for: Ecological Implications of a Flower Size/Number Trade-Off in Tropical Forest Trees
Source: PLoS One. 2011 Feb 1;6(2):e16111. doi: 10.1371/journal.pone.0016111 (PMC3052255; doi:10.1371/journal.pone.0016111)
Supplement: Table S2 — Mating system statistics for progeny of Shorea xanthophylla and Parashorea tomentella based upon 9 microsatellites loci. Number of progeny genotypes (N); multilocus outcrossing rate (tm); single locus outcrossing rate (ts); biparental inbreeding as defined by the difference between multilocus and single locus outcrossing rates (tm - ts); Parental inbreeding coefficient. Values in parentheses are standard error (SE) based upon 100 bootstraps. (DOCX) [file pone.0016111.s003.docx]

**Table S2**. Mating system statistics for progeny of *Shorea xanthophylla* and *Parashorea tomentella* based upon 9 microsatellites loci. Number of progeny genotypes (*N*); multilocus outcrossing rate (*t_m_* ); single locus outcrossing rate (*t_s_*); , biparental inbreeding as defined by the difference between multilocus and single locus outcrossing rates (*t_m_ - t_s_*); Parental inbreeding coefficient. Values in parenthesese are standard error (SE) based upon 100 bootstraps.

|  | *N* | *t_m_* | *t_s_* | *t_m_ - t_s_* | *F_p_* |
| --- | --- | --- | --- | --- | --- |
| *S.xanthophylla* | 456 | 0.996 | 0.9 | 0.096 | 0.094 |
|  |  | (0.0097) | (0.0022) | (0.0102) | (0.0046) |
| *P.tomentella* | 408 | 0.907 | 0.854 | 0.053 | -0.031 |
|  |  | (0.0014) | (0.0008) | (0.0007) | (0.0018) |
