# Supplementary material for: Ecological Implications of a Flower Size/Number Trade-Off in Tropical Forest Trees
Source: PLoS One. 2011 Feb 1;6(2):e16111. doi: 10.1371/journal.pone.0016111 (PMC3052255; doi:10.1371/journal.pone.0016111)
Supplement: Table S3 — Summary of the 11 microsatellite primers used for paternity analysis and quantification of relatedness between assigned parents in two dipterocarp species Shorea xanthophylla and Parashorea tomentella. Number of alleles (Na); observed heterozygosity (Hobs); expected heterozygosity (H e); paternity non-exclusion probability at each locus (N-PE) and total exclusion probability over all loci (PE) given known mother. a Redesigned primers based on published primers. b Newly developed microsatellite primers. c Published primers. (DOCX) [file pone.0016111.s004.docx]

Table S3. Summary of the 11 microsatellite primers used for paternity analysis and quantification of relatedness between assigned parents in two dipterocarp species *Shorea xanthophylla* and *Parashorea tomentella.* Number of alleles (Na); observed heterozygosity (*H_obs_*); expected heterozygosity (*H*_e_); paternity non-exclusion probability at each locus (*N-P_E_*) and total exclusion probability over all loci (*P_E_*) given known mother. *^a^* Redesigned primers based on published primers. *^b^* Newly developed microsatellite primers. ^c^ Published primers.

| *S.xanthophylla* | Locus | Na | Size range (bp) | *H_obs_* | *H*_e_ | *N-P_E_* | *GeneBank Accession* |
| --- | --- | --- | --- | --- | --- | --- | --- |
| (108 adults) | *Dip01^a^* | 4 | 89-95 | 0.062 | 0.089* | 0.954 | AJ582737 |
|  | *Dip02 ^a^* | 12 | 206-229 | 0.716 | 0.78 | 0.396 | AJ616883.1 |
|  | *Dip03 ^a^* | 9 | 137-154 | 0.48 | 0.524 | 0.679 | AJ616888.1 |
|  | *Dip04 ^a^* | 10 | 146-190 | 0.295 | 0.568* | 0.683 | AY558717 |
|  | *Dip05 ^a^* | 9 | 175-192 | 0.621 | 0.694 | 0.514 | AJ616885 |
|  | *Pt05 ^b^* | 6 | 103-117 | 0.688 | 0.694 | 0.559 | FJ968736 |
|  | *SLD1^c^* | 15 | 181-212 | 0.839 | 0.809 | 0.358 | DC651058 |
|  | *Sx02 ^a^* | 27 | 200-253 | 0.795 | 0.876 | 0.238 | FJ968737 |
|  | *Sx10 ^a^* | 19 | 121-179 | 0.897 | 0.889 | 0.217 | FJ968738 |
|  | **mean** | **12** |  | **0.599** | **0.658** | **0.511** |  |
|  | ***P_E_*** |  |  |  |  | 0.9991 |  |
| *P.tomentella* | Locus | Na | Size range (bp) | *H_obs_* | *H*_e_ | *N-P_E_* |  |
| (93 adults) | *Dip01^a^* | 6 | 102-116 | 0.506 | 0.519 | 0.7 | AJ582737 |
|  | *Dip02 ^a^* | 10 | 212-240 | 0.75 | 0.781 | 0.425 | AJ616883.1 |
|  | *Dip03 ^a^* | 7 | 134-146 | 0.231 | 0.312* | 0.839 | AJ616888.1 |
|  | *Dip04 ^a^* | 6 | 158-172 | 0.659 | 0.741 | 0.501 | AY558717 |
|  | *Dip05 ^a^* | 7 | 178-198 | 0.368 | 0.428 | 0.752 | AJ616885 |
|  | *Pt05 ^a^* | 10 | 97-119 | 0.651 | 0.785 | 0.419 | FJ968736 |
|  | *SLD1^c^* | 7 | 189-237 | 0.194 | 0.368* | 0.792 | DC651058 |
|  | *SLK06 ^c^* | 5 | 144-156 | 0.627 | 0.567 | 0.726 | DC649188 |
|  | *SLC06 ^c^* | 12 | 202-224 | 0.667 | 0.789 | 0.391 | DC650703 |
|  | **mean** | **8** |  | **0.517** | **0.588** | **0.616** |  |
|  | ***P_E_*** |  |  |  |  | 0.9912 |  |

**Table S3**. Mating system statistics for progeny of *Shorea xanthophylla* and *Parashorea tomentella* based upon 9 microsatellites loci. Number of progeny genotypes (*N*); multilocus outcrossing rate (*t_m_* ); single locus outcrossing rate (*t_s_*); , biparental inbreeding as defined by the difference between multilocus and single locus outcrossing rates (*t_m_ - t_s_*); Parental inbreeding coefficient. Values in parenthesese are standard error (SE) based upon 100 bootstraps.

|  | *N* | *t_m_* | *t_s_* | *t_m_ - t_s_* | *F_p_* |
| --- | --- | --- | --- | --- | --- |
| *S.xanthophylla* | 456 | 0.996 | 0.9 | 0.096 | 0.094 |
|  |  | (0.0097) | (0.0022) | (0.0102) | (0.0046) |
| *P.tomentella* | 408 | 0.907 | 0.854 | 0.053 | -0.031 |
|  |  | (0.0014) | (0.0008) | (0.0007) | (0.0018) |
